# 

**Published:** 1897-09-11

**Authors:** 


### EARLY EDITION.

**Notices of Births, Deaths, and Marriages** are inserted in this column at a charge of 2s. 6d. for an announcement not exceeding four lines.

### NOTICE TO CORRESPONDENTS.

All MS., Letters, Books ror Review, and other matters intended for the Editor should be addressed The Editor, "The Hospital," The Hospital Building, 28 & 29, Southampton Street, Strand, W.C.

The Editor cannot undertake to return rejected MS., even when accompanied by stamped directed envelope.

**Notice to Advertisers and Subscribers.**

All Advertisements, Orders for Copies of The Hospital, and Business Communications should be addressed to **THE MANAGER, (Not to The Editor),** The Hospital, The Hospital Building, 28 & 29, Southampton Street, Strand, London, W.C.

The Cost of Subscription, post free, is as follows:—Per week, 2½d.; per quarter, 2s. 8d.; per half-year, 5s. 3d.; per annum, 10s. 6d. Foreign:—Per annum, 15s. 2d.; payable, in all cases, in advance.

**NOTICE.—Vol. XXI. of "The Hospital" (October, 1896, to March, 1897), in handsome cloth binding, is on sale at the Office of this Journal, price 7s. 6d. Cases for binding the Numbers contained in this Volume, 1s. 6d. Index to Vol. XXI., 2½d., post free.**

**The Monthly Part for September is now ready, Price 6d., by post 8d.**

### BOOKS RECEIVED.

"The Vegetarian" Office.

"Flesh Eating a Cause of Consumption." Second Edition. By Josiah Oldfield, M.A., B.C.L.

The Open Court Publishing Co.

"The Open Court Monthly Magazine."

J. B. Lippincott Company.

"System of Diseases of the Eye." Vol. II. By American, British, Dutch, French, German, and Spanish authors.

"Lippincott's Medical Dictionary." By Ryland W. Greene, A.B.

Edinburgh: E. and S. Livingstone. Glasgow: N. Adshead and Son.

"The Insane in Private Dwellings and Licensed Houses." By J. F. Sutherland, M.D.F.R.S.E. Second Edition.

Swan Sonnenschein and Co.

"A Bibliography of Medicine." By William Swan Sonnenschein.

Simpkin, Marshall, Hamilton, Kent, and Co.

"The A B C of the X-Rays." By William H. Meadowcroft.

### The Student of Medicine.

### THE LONDON HOSPITAL MEDICAL SCHOOL.

### Important to Students.

No pains have been spared to keep this school in every respect abreast of modern requirements. It has, as we said last week, the largest clinical field, owing to the immense number of accident cases, and in other ways the material for clinical study is unrivalled. We are informed that the teaching will be enormously improved this year. A new rule has been passed giving the assistant surgeons 20 beds each, and fixing a day each week when each assistant surgeon must teach the students at the bedside. In this way students will have the advantage of the experience of the senior members of the staff, and also that of young, keen, energetic men, who must rely upon their success in this direction to secure a position for themselves. This is a new departure worthy of commendation, and must prove beneficial, not only to the patients, but to the students and the London Hospital Medical School. A bacteriologist has also been appointed, who will devote his whole time to the work, and numerous other improvements are being introduced at a cost of £2,000 a year to the school. It follows that the teaching and scientific opportunities afforded at the London Hospital will equal, if they do not even surpass, those of any other metropolitan school.

### APPOINTMENTS.

F. W. Abbott, L.R.C.P.Lond., M.R.C.S.Eng., appointed Resident Obstetric Physician to Charing Cross Hospital. W. F. Adams, M.R.C.S.Eng., L.R.C.P.Lond., appointed Senior Assistant Medical Officer to the Cornwall County Lunatic Asylum at Bodmin. G. M. Arkle, L.S.A., appointed Medical Officer of the North Part of the No. 2 District of the West Derby Union. C. W. R. Banham, M.R.C.S., L.R.C.P., appointed Medical Officer of the No. 8 District of the Weymouth Union. H. S. Beadles, M.R.C.S.Eng., L.R.C.P.Lond., appointed House Surgeon to the Grimsby and District Hospital. J. Clay, M.B., B.S., appointed Surgical Registrar to the Royal Infirmary, Newcastle-on-Tyne. E. F. Clowes, M.R.C.S.Eng., L.R.C.P.Lond., appointed House Surgeon to the Royal Hants County Hospital, Winchester. R. P. Crosbie, M.B., B.Ch., appointed Extern Surgeon to the South Charitable Infirmary, Cork. G. E. Crowe, B.A.Dub., M.D., B.Ch., appointed Medical Officer of the No. 2 District of the Chorlton Union. B. G. Ewing, M.B., C.M.Edin., appointed Medical Officer of Health to the Ardsley East and West Urban District. W. R. Fenton, B.A., M.B.Dub., appointed Police Surgeon at Salisbury, Mashonaland. R. B. Ferguson, M.A., M.D.Cantab., appointed Surgeon to the Y Division of Police at New Southgate. J. F. Fernie, M.R.C.S., L.R.C.P., appointed House Surgeon to the Staffordshire General Infirmary. T. Gibson, M.B., C.M.Edin., appointed Resident Assistant Medical Officer of the Birkenhead Union Workhouse. C. Goring, M.R.C.S., L.R.C.P., appointed Senior House Physician to the Great Northern Central Hospital, London. B. C. Gowing, M.R.C.S.Eng., L.S.A., appointed Medical Officer of the No. 5 District of the Wortley Union. W. Graham, M.D., R.U.I., Medical Superintendent, Armagh County Asylum, appointed Medical Superintendent to the Belfast District Lunatic Asylum. A. Greenwood, M.B., L.R.C.P.E., appointed Medical Officer of the Salford Union Infirmary. Mary B. Hannay, M.B., C.M.-Glasg., appointed Medical Officer for the Islands of Flotta and Flara, Orkney. J. Hartley, M.B., Ch.B.Vict., appointed House Surgeon to Ancoats Hospital, Manchester. A. Heath, M.B.Lond., M.R.C.S., L.R.C.P., appointed House Physician to St. Bartholomew's Hospital. W. Hirst, L.R.C.P.Edin., L.R.C.S., appointed Medical Officer of the Howden District of the Auckland Union. A. D. Howard, B.A., M.D.Brux., appointed Medical Officer of the New Hampton District of the Kingston Union. H. W. M. Kendall, M.R.C.S.Eng., appointed House Surgeon to the Central London Ophthalmic Hospital. W. W. Leigh, L.R.C.P.Edin.,&L.M., M.R.C.S.Eng., L.S.A., appointed Medical Officer and Public Vaccinator to the District of Llanfabon.

St. Thomas's Hospital.—The following gentlemen have been selected as House Officers from September 7th, 1897:—House Physicians: H. C. Jonas, C. G. Seligmann, W. McDougall, and H. N. Goode. House Surgeons: W. H. J. Paterson, A. W. Tuke, L. Gilbert, and S. N. Babington. Assistant House Surgeons: J. F. McClean, H. J. Marriage, J. S. Hall, and H. H. Sanguinetti. Obstetric House Physicians: J. S. Fairbairn (Senior), G. D. Hindley (Junior). Ophthalmic House Surgeons: E. Hopkinson (Senior), F. A. C. Tyrrell (Junior). Clinical Assistants in the Throat Department: J. A. A. Rouillard and W. Myers. Clinical Assistants in the Skin Department: E. Stainer and E. H. Cobb. Clinical Assistants in the Ear Department: W. D. Frazer and S. D. Turner.

